# Supplementary material for: Longitudinal Circulating Tumor DNA Analysis in Blood and Saliva for Prediction of Response to Osimertinib and Disease Progression in EGFR-Mutant Lung Adenocarcinoma
Source: Cancers (Basel). 2021 Jul 3;13(13):3342. doi: 10.3390/cancers13133342 (PMC8268167; doi:10.3390/cancers13133342)
Supplement: Supplementary file 1 [file cancers-13-03342-s001.zip › Supplemental tables Kim et al Cancers.pdf]

Supplementary Table S1. Patient characteristics.

**Median age**		60 (range: 30-77)
**Sex**	Female	18 (72%)
	Male	7 (28%)
**Race**	White	14 (56%)
	Asian	5 (20%)
	African American	5 (20%)
	Multirace	1 (4%)
**Histology**	Adenocarcinoma	25 (100%)
***EGFR* mutations**	Exon 19 deletion	10 (40%)
	L858R	4 (16%)
	Exon 19 deletion/T790M	8 (32%)
	Exon L8585R/T790M	2 (8%)
	Exon 20 insertion/V769L	1 (4%)
**Cohort**	1	15 (60%)
	2	7 (28%)
	3	3 (12%)

Supplementary Table S2. List of *EGFR* mutations detected by ddPCR.

Amino Acid Change	Nucleotide Mutation	Chromosomal Location (hg19)	Amplicon Length (bp)	COSMIC ID	Mutation Description
p.E746_A750delELREA	c.2235_2249del15	chr7:55242404-55242540	78	COSM6223	Deletion - In frame
p.E746_A750delELREA	c.2236_2250del15	chr7:55242405-55242541	91	COSM6225	Deletion - In frame
p.E746_S752>V	c.2237_2256>TC	chr7:55242406-55242547	99	COSM18426	Complex - deletion inframe
p.L747_A750>P	c.2239_2248TTAAGAGAAG>C	chr7:55242408-55242539	84	COSM12382	Complex - deletion inframe
p.V769L	c.2305G>C	chr7:55248946-55249068	59	NA	Substitution - Missense
p.T790M	c.2369C>T	chr7:55249010-55249132	80	COSM6240	Substitution - Missense
p.L858R	c.2573T>G	chr7:55259454-55259576	73	COSM6224	Substitution - Missense

Supplementary Table S3. 36 genes included in InvisionFirst™-Lung

AKT1	ALK	BRAF	CCND1	CDKN2A	CTNNB1
EGFR	ERBB2	ESR1	FGFR1	FGFR2	FGFR3
GATA3	GNA11	GNAQ	GNAS	HRAS	IDH1
IDH2	KIT	KRAS	MAP2K1	MET	MYC
NFE2L2	NRAS	NTRK1	NTRK3	PDGFRA	PIK3CA
PPP2R1A	PTEN	ROS1	STK11	TP53	U2AF1

SNVs + Indels - Hotspot Regions

Fusions + SNVs + Indels

CNVs only

CNVs + SNVs + Indels

SNVs + Indels - Exon Coverage 70% for PTEN, 88-100% for TP53, STK11 and CDKN2A

Supplementary Table S4. Probe sequences: Capture probe and detector probe oligonucleotide sequences for three TKI-sensitive mutations. The detector probes are biotinylated at the 3’ end.

	**Capture Probe Sequences**	**Detector Probe Sequences**
Exon 19 deletion	5’ – TGTTGCTTCCTTG – 3’	5’ – ATAGCGACGGGAATTTTAACTTTCTCACCT – 3’
p.L858R	5’ – GTTTGACCCGCCCA – 3’	5’ – AAAATCTGTGATCTTGACATGCTGCGGTGTTTTGTGCAG – 3’
p.T790M	5’ – GAGCGGCATGATGA – 3’	5’ – GCTGCACGGTGGAGGTGAGGCAGATGCCCAGC – 3’

Supplementary Table S5. Number and sites of lesions measured by volumetric measurements.

LAT	CT Scan (time point)	Total Lesion	Pulmonary	Liver	Node	Other
001	8	26	1	25	0	0
002	9	2	1	0	1	0
003	11	3	1	2	0	0
004	14	1	1	0	0	0
005	4	5	2	0	1	2
006	10	10	5	0	4	1
007	17	1	1	0	0	0
008	13	3	1	1	1	0
009	12	3	1	0	0	2
010	13	4	1	1	2	0
011	12	6	3	2	0	1
013	4	3	1	2	0	0
014	13	3	2	1	0	0
015	5	2	2	0	0	0
016	19	4	1	1	0	2
017	5	6	1	0	1	4
019	9	1	1	0	0	0
020	6	2	2	0	0	0
021	12	6	1	0	2	3
022	14	3	3	0	0	0
023	13	2	1	0	1	0
024	11	2	2	0	0	0
025	12	5	2	0	3	0
026	13	3	1	0	1	1
028	6	6	2	0	1	3
Average	10.6	4.48	1.6	1.4	0.72	0.76
Min	4	1	1	0	0	0
Max	19	26	5	25	4	4
total	265	112	40	35	18	19
Median	12	3	1	0	0	0

Supplementary Table S6. Spearman correlation between allele frequencies of sensitizing *EGFR* mutations by ddPCR and NGS, current values (nA) of eLB, and calculated tumor volume.

	ddPCR	NGS	eLB	Calculated tumor volume
ddPCR	1	0.93*	0.24*	0.35*
NGS	0.93*	1	0.24**	0.46*
eLB	0.24*	0.24**	1	0.28*
Total tumor volume	035*	0.46*	0.28*	1

* P value <0.001 (test of r=0)
** P value <0.05
