# Supplementary material for: Longitudinal Circulating Tumor DNA Analysis in Blood and Saliva for Prediction of Response to Osimertinib and Disease Progression in EGFR-Mutant Lung Adenocarcinoma
Source: Cancers (Basel). 2021 Jul 3;13(13):3342. doi: 10.3390/cancers13133342 (PMC8268167; doi:10.3390/cancers13133342)

Supplementary Figure legends

Supplementary Figure S1. Multi-Planar Volume Rendered (MPVR) images show the volume and shape of a lung lesion in LAT011 and multiple liver lesions in LAT001. Baseline axial (A) and coronal (B) images, versus follow up axial (C) and coronal (D) images at follow up.

Supplementary Figure S2. Patients without ctDNA progression prior to RECIST 1.1 progression.

Supplementary Figure S3. Patients with no RECIST 1.1 progression.

Supplementary Figure S1. Assessment of tumor volume by volumetric CT measurement.

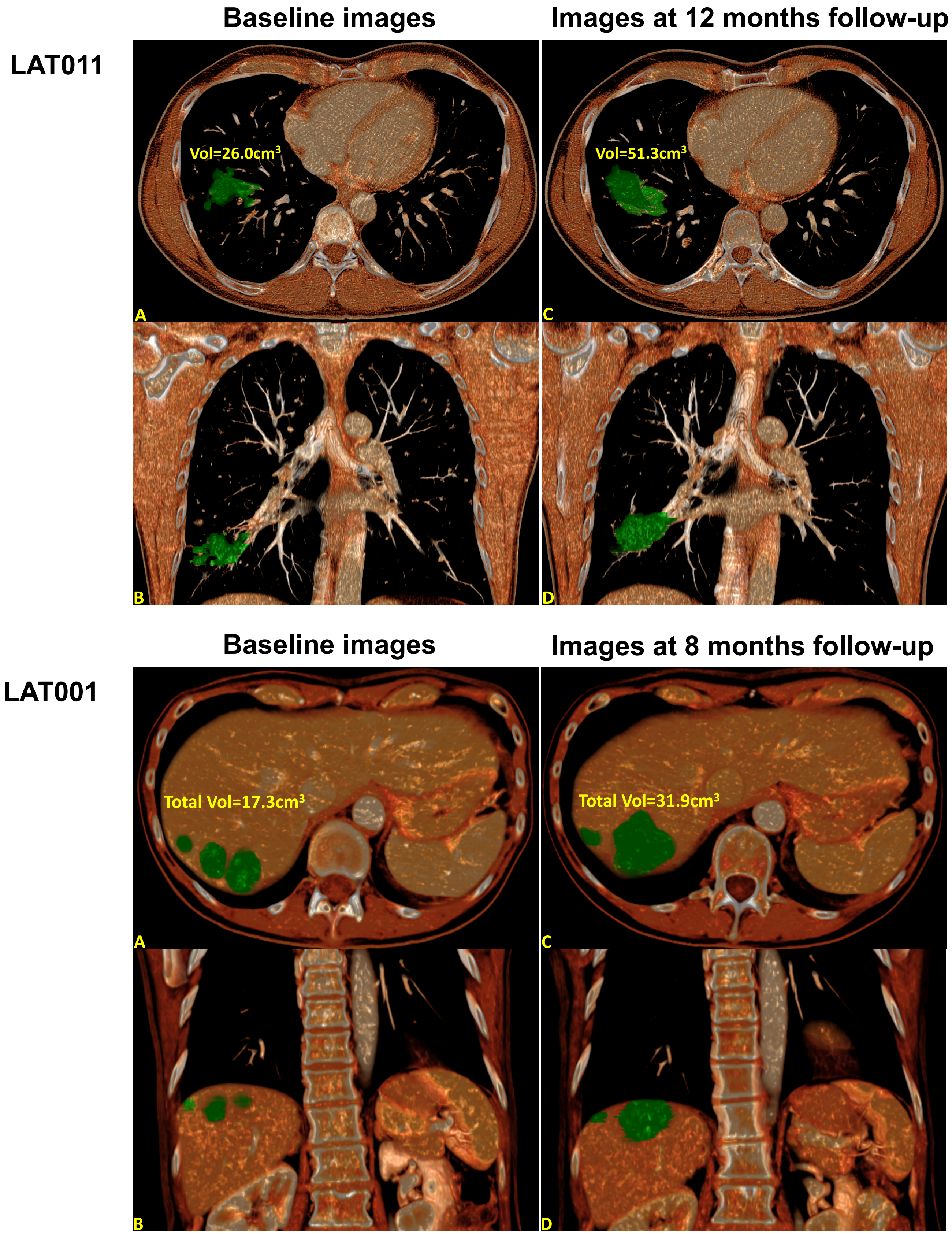

Supplementary Figure S2. Patients without ctDNA progression prior to RECIST progression.

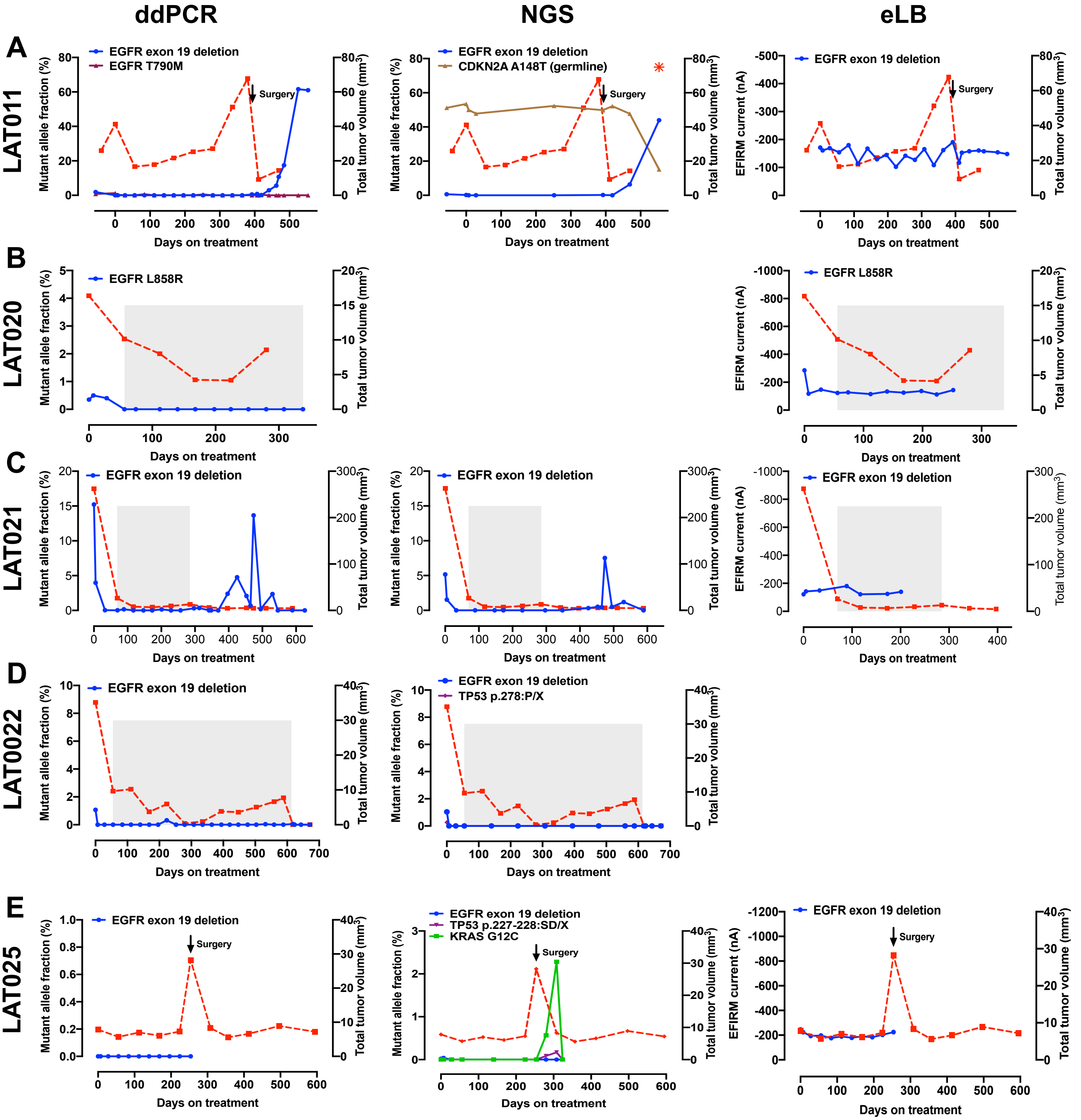

Supplementary Figure S3. Patients with no RECIST progression.

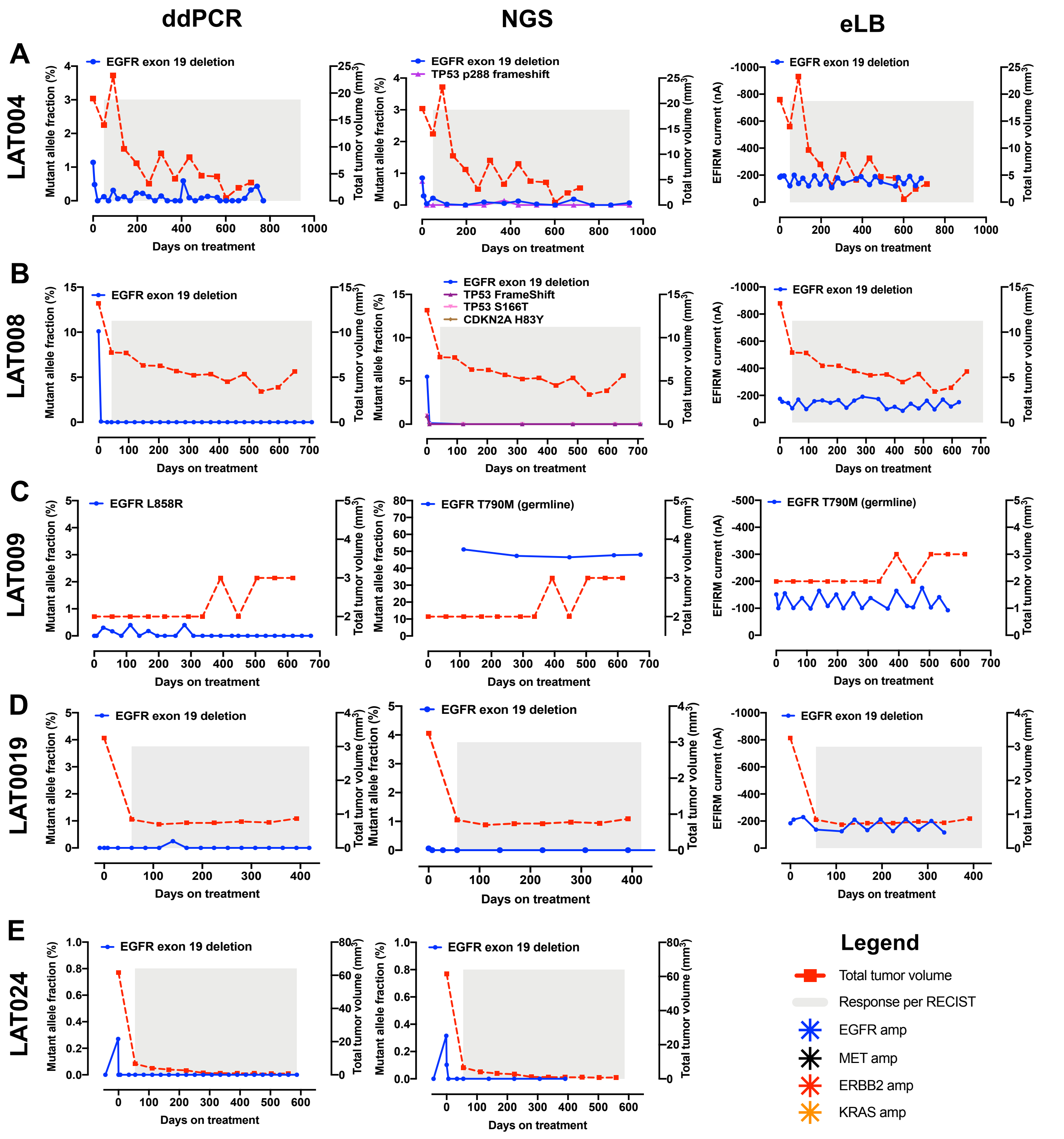

Supplement: Supplementary file 1 [file cancers-13-03342-s001.zip › Supplementary Figures_Kim et al.pdf]
