# Supplementary material for: Longitudinal Circulating Tumor DNA Analysis in Blood and Saliva for Prediction of Response to Osimertinib and Disease Progression in EGFR-Mutant Lung Adenocarcinoma
Source: Cancers (Basel). 2021 Jul 3;13(13):3342. doi: 10.3390/cancers13133342 (PMC8268167; doi:10.3390/cancers13133342)
Supplement: Supplementary file 1 [file cancers-13-03342-s001.zip › Supplementary Table S7.pdf]

Patient ID	Collection Date	TxDay	Cycle	Code	Gene name	Protein change	Consequence	Cosmic name	Frequency percentage
LAT001	2016-04-13	-7	Pre	del_chr17_7675997_GCAAGTCACAGACTTGGCTGT_-	TP53	p.118-124:TAKSVTC/-	inframe_deletion	NA	16.5241
				del_chr7_55174772_GGAATTAAGAGAAGC_-	EGFR	p.745-750:KELREA/K	inframe_deletion	COSM6223	49.1711
				amplification_KRAS	KRAS	NA	NA	NA	55.5749
				mis_chr3_179234289_T_G	PIK3CA	p.N1044K	missense_variant	COSM27504	31.2968
	2016-04-20	0	C1D0	del_chr17_7675997_GCAAGTCACAGACTTGGCTGT_-	TP53	p.118-124:TAKSVTC/-	inframe_deletion	NA	0.8451
				del_chr7_55174772_GGAATTAAGAGAAGC_-	EGFR	p.745-750:KELREA/K	inframe_deletion	COSM6223	2.5880
				mis_chr3_179234289_T_G	PIK3CA	p.N1044K	missense_variant	COSM27504	1.1620
	2016-07-06	84	C5D1	mis_chr7_55191822_T_G	EGFR	p.L858R	missense_variant	COSM6224	0.1563
	2016-09-07	147	C8D1	del_chr7_55174772_GGAATTAAGAGAAGC_-	EGFR	p.745-750:KELREA/K	inframe_deletion	COSM6223	0.1651
				mis_chr7_55181378_C_T	EGFR	p.T790M	missense_variant	COSM6240	0.9906
				mis_chr7_55191822_T_G	EGFR	p.L858R	missense_variant	COSM6224	0.4953
	2016-09-28	168	C9D1	del_chr17_7675997_GCAAGTCACAGACTTGGCTGT_-	TP53	p.118-124:TAKSVTC/-	inframe_deletion	NA	0.1980
				del_chr7_55174772_GGAATTAAGAGAAGC_-	EGFR	p.745-750:KELREA/K	inframe_deletion	COSM6223	5.6188
				mis_chr3_179234289_T_G	PIK3CA	p.N1044K	missense_variant	COSM27504	0.6188
	2016-11-09	210	C11D1	amplification_EGFR	EGFR	NA	NA	NA	NA
				amplification_MET	MET	NA	NA	NA	NA
				del_chr17_7675997_GCAAGTCACAGACTTGGCTGT_-	TP53	p.118-124:TAKSVTC/-	inframe_deletion	NA	5.6604
				del_chr7_55174772_GGAATTAAGAGAAGC_-	EGFR	p.745-750:KELREA/K	inframe_deletion	COSM6223	46.8160
				amplification_KRAS	KRAS	NA	NA	NA	53.7028
				mis_chr3_179234289_T_G	PIK3CA	p.N1044K	missense_variant	COSM27504	18.8679
	2016-11-29	230	ReTxC1D0	del_chr7_55174772_GGAATTAAGAGAAGC_-	EGFR	p.745-750:KELREA/K	inframe_deletion	COSM6223	0.0250
	2017-01-04	266	ReTxC2D7	del_chr17_7675997_GCAAGTCACAGACTTGGCTGT_-	TP53	p.118-124:TAKSVTC/-	inframe_deletion	NA	2.7869
				del_chr7_55174772_GGAATTAAGAGAAGC_-	EGFR	p.745-750:KELREA/K	inframe_deletion	COSM6223	12.1175
				mis_chr3_179234289_T_G	PIK3CA	p.N1044K	missense_variant	COSM27504	4.9590
	2017-01-25	287	ReTxC3D1	amplification_MET	MET	NA	NA	NA	NA
				del_chr17_7675997_GCAAGTCACAGACTTGGCTGT_-	TP53	p.118-124:TAKSVTC/-	inframe_deletion	NA	16.8625
				del_chr7_55174772_GGAATTAAGAGAAGC_-	EGFR	p.745-750:KELREA/K	inframe_deletion	COSM6223	53.5250
				amplification_KRAS	KRAS	NA	NA	NA	NA
				mis_chr3_179234289_T_G	PIK3CA	p.N1044K	missense_variant	COSM27504	36.2500
LAT002	2016-05-11	0	C1D0	mis_chr7_55191822_T_G	EGFR	p.L858R	missense_variant	COSM6224	0.5978
	2016-05-18	7	C1D7	No call for this sample	NA	NA	NA	NA	NA
	2016-08-10	91	C5D1	mis_chr7_55191822_T_G	EGFR	p.L858R	missense_variant	COSM6224	0.0917
	2017-01-25	259	C12D1	mis_chr7_55191822_T_G	EGFR	p.L858R	missense_variant	COSM6224	1.0058
	2017-03-22	315	C14D1	mis_chr3_179218303_G_A	PIK3CA	p.E545K	missense_variant	COSM763	0.0641
				mis_chr7_55191822_T_G	EGFR	p.L858R	missense_variant	COSM6224	9.9840
	2017-04-19	343	C15D1	mis_chr7_55191822_T_G	EGFR	p.L858R	missense_variant	COSM6224	2.5272
	2017-05-12	366	Off	mis_chr7_55191822_T_G	EGFR	p.L858R	missense_variant	COSM6224	2.9000
	2017-07-31	446	Off	mis_chr7_55191822_T_G	EGFR	p.L858R	missense_variant	COSM6224	3.9273
LAT003	2016-06-01	0	C1D0	del_chr7_55174772_GGAATTAAGAGAAGC_-	EGFR	p.745-750:KELREA/K	inframe_deletion	COSM6223	48.1510
				mis_chr17_7675051_A_G	TP53	NA	splice_donor_variant	NA	16.8620
				mis_chr7_55181378_C_T	EGFR	p.T790M	missense_variant	COSM6240	11.1133
	2016-06-08	7	C1D7	amplification_ERBB2	ERBB2	NA	NA	NA	NA
				del_chr7_55174772_GGAATTAAGAGAAGC_-	EGFR	p.745-750:KELREA/K	inframe_deletion	COSM6223	7.5250
				mis_chr17_7675051_A_G	TP53	NA	splice_donor_variant	NA	2.3750
				mis_chr7_55181378_C_T	EGFR	p.T790M	missense_variant	COSM6240	1.0000
	2016-12-14	196	C10D1	del_chr7_55174772_GGAATTAAGAGAAGC_-	EGFR	p.745-750:KELREA/K	inframe_deletion	COSM6223	11.1390
				mis_chr17_7675051_A_G	TP53	NA	splice_donor_variant	NA	5.5019
				mis_chr3_179218303_G_A	PIK3CA	p.E545K	missense_variant	COSM763	0.1255
	2017-04-07	310	ReTxC4D1	del_chr7_55174772_GGAATTAAGAGAAGC_-	EGFR	p.745-750:KELREA/K	inframe_deletion	COSM6223	14.2250
				mis_chr17_7675051_A_G	TP53	NA	splice_donor_variant	NA	13.9375
				mis_chr3_179218303_G_A	PIK3CA	p.E545K	missense_variant	COSM763	0.1500
				mis_chr7_55181378_C_T	EGFR	p.T790M	missense_variant	COSM6240	0.4000
	2017-06-21	385	ReTxC4D1	del_chr7_55174772_GGAATTAAGAGAAGC_-	EGFR	p.745-750:KELREA/K	inframe_deletion	COSM6223	35.0787
				mis_chr17_7675051_A_G	TP53	NA	splice_donor_variant	NA	4.0748
				mis_chr7_55181378_C_T	EGFR	p.T790M	missense_variant	COSM6240	5.4823
				mis_chr7_55181399_G_C	EGFR	p.C797S	missense_variant	NA	2.0571
	2017-08-16	441	ReTxC6D1	amplification_EGFR	EGFR	NA	NA	NA	NA
				amplification_ERBB2	ERBB2	NA	NA	NA	NA
				del_chr7_55174772_GGAATTAAGAGAAGC_-	EGFR	p.745-750:KELREA/K	inframe_deletion	COSM6223	74.1125
				mis_chr17_7675051_A_G	TP53	NA	splice_donor_variant	NA	22.6500
				mis_chr7_55181378_C_T	EGFR	p.T790M	missense_variant	COSM6240	13.9750
				mis_chr7_55181399_G_C	EGFR	p.C797S	missense_variant	NA	4.1250
	2017-08-30	455	ReTxC6D14	amplification_EGFR	EGFR	NA	NA	NA	NA
				del_chr7_55174772_GGAATTAAGAGAAGC_-	EGFR	p.745-750:KELREA/K	inframe_deletion	COSM6223	58.3063
				mis_chr17_7675051_A_G	TP53	NA	splice_donor_variant	NA	22.3375
				mis_chr3_179218303_G_A	PIK3CA	p.E545K	missense_variant	COSM763	0.1125
				mis_chr7_55181378_C_T	EGFR	p.T790M	missense_variant	COSM6240	11.6563
				mis_chr7_55181399_G_C	EGFR	p.C797S	missense_variant	NA	3.4250
	2017-10-23	509	Off	amplification_EGFR	EGFR	NA	NA	NA	NA
				del_chr7_55174772_GGAATTAAGAGAAGC_-	EGFR	p.745-750:KELREA/K	inframe_deletion	COSM6223	81.3125
				mis_chr17_7675051_A_G	TP53	NA	splice_donor_variant	NA	21.1000
				mis_chr7_55181378_C_T	EGFR	p.T790M	missense_variant	COSM6240	15.1375
				mis_chr7_55181399_G_C	EGFR	p.C797S	missense_variant	NA	5.3000

LAT004	2016-07-13	0	C1D0	del_chr17_7673758_T_-	TP53	p.288:N/X	frameshift_variant	COSM45459	0.8537
				del_chr7_55174772_GGAATTAAGAGAAGC_-	EGFR	p.745-750:KELREA/K	inframe_deletion	COSM6223	0.7520
	2016-07-20	7	C1D7	del_chr17_7673758_T_-	TP53	p.288:N/X	frameshift_variant	COSM45459	0.3237
				del_chr7_55174772_GGAATTAAGAGAAGC_-	EGFR	p.745-750:KELREA/K	inframe_deletion	COSM6223	0.2878
	2016-08-03	21	C2D1	del_chr7_55174772_GGAATTAAGAGAAGC_-	EGFR	p.745-750:KELREA/K	inframe_deletion	COSM6223	0.0435
	2016-08-31	49	C3D1	del_chr7_55174772_GGAATTAAGAGAAGC_-	EGFR	p.745-750:KELREA/K	inframe_deletion	COSM6223	0.2167
	2016-11-02	112	C6D1	del_chr7_55174772_GGAATTAAGAGAAGC_-	EGFR	p.745-750:KELREA/K	inframe_deletion	COSM6223	0.0249
	2017-01-25	196	C9D1	No call for this sample	NA	NA	NA	NA	
	2017-04-19	280	C12D1	del_chr7_55174772_GGAATTAAGAGAAGC_-	EGFR	p.745-750:KELREA/K	inframe_deletion	COSM6223	0.0943
	2017-07-19	371	C15D1	del_chr17_7673758_T_-	TP53	p.288:N/X	frameshift_variant	COSM45459	0.1418
				del_chr7_55174772_GGAATTAAGAGAAGC_-	EGFR	p.745-750:KELREA/K	inframe_deletion	COSM6223	0.0532
	2017-09-21	435	C17D1	del_chr7_55174772_GGAATTAAGAGAAGC_-	EGFR	p.745-750:KELREA/K	inframe_deletion	COSM6223	0.1276
	2017-12-13	518	C20D1	del_chr7_55174772_GGAATTAAGAGAAGC_-	EGFR	p.745-750:KELREA/K	inframe_deletion	COSM6223	0.0305
	2018-03-07	602	C23D1	No call for this sample	NA	NA	NA	NA	NA
	2018-05-30	686	C26D1	del_chr7_55174772_GGAATTAAGAGAAGC_-	EGFR	p.745-750:KELREA/K	inframe_deletion	COSM6223	0.1875
	2018-08-22	770	C29D1	No call for this sample	NA	NA	NA	NA	NA
	2018-11-14	854	C32D1	No call for this sample	NA	NA	NA	NA	NA
	2019-02-06	938	C35D1	del_chr7_55174772_GGAATTAAGAGAAGC_-	EGFR	p.745-750:KELREA/K	inframe_deletion	COSM6223	0.0688
LAT005	2016-08-17	0	ReTxC1D0	mis_chr17_7674180_C_T	TP53	NA	splice_donor_variant	NA	19.4459
				mis_chr7_55191822_T_G	EGFR	p.L858R	missense_variant	COSM6224	14.3879
	2016-08-24	7	ReTxC1D7	amplification_ERBB2	ERBB2	NA	NA	NA	NA
				mis_chr17_7674180_C_T	TP53	NA	splice_donor_variant	NA	21.2406
				mis_chr7_55191822_T_G	EGFR	p.L858R	missense_variant	COSM6224	17.5627
	2016-09-07	21	ReTxC2D1	amplification_EGFR	EGFR	NA	NA	NA	NA
				amplification_ERBB2	ERBB2	NA	NA	NA	NA
				mis_chr17_7674180_C_T	TP53	NA	splice_donor_variant	NA	17.1068
				mis_chr7_55181378_C_T	EGFR	p.T790M	missense_variant	COSM6240	0.1484
				mis_chr7_55191822_T_G	EGFR	p.L858R	missense_variant	COSM6224	15.1484
	2016-09-28	42	ReTxC3D1	mis_chr17_7674180_C_T	TP53	NA	splice_donor_variant	NA	38.6007
				mis_chr7_55191822_T_G	EGFR	p.L858R	missense_variant	COSM6224	26.4249
	2016-10-26	70	ReTxC4D1	amplification_ERBB2	ERBB2	NA	NA	NA	NA
				mis_chr17_7674180_C_T	TP53	NA	splice_donor_variant	NA	38.5625
				mis_chr7_55191822_T_G	EGFR	p.L858R	missense_variant	COSM6224	21.0875
	2017-06-16	303	Off	mis_chr17_7674180_C_T	TP53	NA	splice_donor_variant	NA	58.7875
				mis_chr7_55191822_T_G	EGFR	p.L858R	missense_variant	COSM6224	29.8188
LAT006	2016-08-03	0	C1D0	amplification_EGFR	EGFR	NA	NA	NA	NA
				del_chr17_7676180_A_-	TP53	p.63:A/X	frameshift_variant	NA	5.8523
				mis_chr7_55181378_C_T	EGFR	p.T790M	missense_variant	COSM6240	0.1847
				mis_chr7_55191822_T_G	EGFR	p.L858R	missense_variant	COSM6224	47.1733
	2016-08-10	7	C1D7	del_chr17_7676180_A_-	TP53	p.63:A/X	frameshift_variant	NA	1.1468
				mis_chr3_41224645_T_C	CTNNB1	p.S45P	missense_variant	COSM5663	0.1262
				mis_chr7_55181378_C_T	EGFR	p.T790M	missense_variant	COSM6240	0.2523
				mis_chr7_55191822_T_G	EGFR	p.L858R	missense_variant	COSM6224	13.7844
	2016-11-15	104	C5D20	mis_chr7_55191822_T_G	EGFR	p.L858R	missense_variant	COSM6224	0.7627
	2017-01-11	161	ReTxC1D7	mis_chr7_55191822_T_G	EGFR	p.L858R	missense_variant	COSM6224	0.0448
	2017-03-01	210	ReTxC3D1	del_chr17_7676180_A_-	TP53	p.63:A/X	frameshift_variant	NA	2.7692
				mis_chr7_55191822_T_G	EGFR	p.L858R	missense_variant	COSM6224	10.3846
	2017-06-19	320	Off	del_chr10_87864493_CGT_-	PTEN	p.8-9:IV/I	inframe_deletion	NA	0.1748
				mis_chr7_55181378_C_T	EGFR	p.T790M	missense_variant	COSM6240	0.1136
				mis_chr7_55191822_T_G	EGFR	p.L858R	missense_variant	COSM6224	1.3287
	2017-12-08	492	Off	del_chr17_7676180_A_-	TP53	p.63:A/X	frameshift_variant	NA	1.2411
				mis_chr7_55191822_T_G	EGFR	p.L858R	missense_variant	COSM6224	11.3653
	2018-04-24	629	Off	No call for this sample	NA	NA	NA	NA	NA
	2018-05-17	652	Off(CSF)	del_chr17_7676180_A_-	TP53	p.63:A/X	frameshift_variant	NA	92.1429
				mis_chr7_55191822_T_G	EGFR	p.L858R	missense_variant	COSM6224	48.9286
	2018-05-21	656	Off(CSF)	del_chr17_7676180_A_-	TP53	p.63:A/X	frameshift_variant	NA	47.7652
				mis_chr7_55191822_T_G	EGFR	p.L858R	missense_variant	COSM6224	30.3599
	2018-05-24	659	Off	No call for this sample	NA	NA	NA	NA	NA
LAT007	2016-08-24	0	C1D0	del_chr7_55174772_GGAATTAAGAGAAGC_-	EGFR	p.745-750:KELREA/K	inframe_deletion	COSM6223	1.8532
				mis_chr10_87933023_T_A	PTEN	p.Y88*	stop_gained	COSM428084	11.6434
	2016-08-31	7	C1D7	del_chr7_55174772_GGAATTAAGAGAAGC_-	EGFR	p.745-750:KELREA/K	inframe_deletion	COSM6223	1.0870
				mis_chr10_87933023_T_A	PTEN	p.Y88*	stop_gained	COSM428084	3.6957
	2016-10-05	42	C3D1	mis_chr10_87933023_T_A	PTEN	p.Y88*	stop_gained	COSM428084	5.2273
	2017-02-08	168	C8D1	No call for this sample	NA	NA	NA	NA	NA
	2017-08-28	369	C15D1	mis_chr10_87933023_T_A	PTEN	p.Y88*	stop_gained	COSM428084	0.6061
	2017-11-21	454	C18D1	No call for this sample	NA	NA	NA	NA	NA
	2018-01-17	511	C20D1	mis_chr10_87933023_T_A	PTEN	p.Y88*	stop_gained	COSM428084	4.9265
	2018-05-04	618	ReTxC1D7	del_chr7_55174772_GGAATTAAGAGAAGC_-	EGFR	p.745-750:KELREA/K	inframe_deletion	COSM6223	0.0132
	2018-05-23	637	ReTxC2D1	del_chr7_55174772_GGAATTAAGAGAAGC_-	EGFR	p.745-750:KELREA/K	inframe_deletion	COSM6223	0.0810
				mis_chr10_87933023_T_A	PTEN	p.Y88*	stop_gained	COSM428084	2.0139
	2018-05-31	645	ReTxC2D1(CSF)	del_chr7_55174772_GGAATTAAGAGAAGC_-	EGFR	p.745-750:KELREA/K	inframe_deletion	COSM6223	58.0688
	2018-06-20	665	ReTxC3D1	mis_chr10_87933023_T_A	PTEN	p.Y88*	stop_gained	COSM428084	0.4875
				mis_chr7_55191802_C_G	EGFR	p.V851V	synonymous_variant	NA	0.1375
	2018-07-17	692	ReTxC4D1	mis_chr10_87933023_T_A	PTEN	p.Y88*	stop_gained	COSM428084	0.4750

	2018-08-15	721	ReTxC5D1	mis_chr10_87933023_T_A	PTEN	p.Y88*	stop_gained	COSM428084	0.3674
	2018-08-17	723	ReTxC5D3(CSF)	del_chr7_55174772_GGAATTAAGAGAAGC_-	EGFR	p.745-750:KELREA/K	inframe_deletion	COSM6223	66.0276
	2018-09-12	749	ReTxC6D1	mis_chr10_87933023_T_A	PTEN	p.Y88*	stop_gained	COSM428084	0.4464
LAT008	2016-09-13	0	C1D0	del_chr7_55174772_GGAATTAAGAGAAGC_-	EGFR	p.745-750:KELREA/K	inframe_deletion	COSM6223	5.4767
				ins_chr17_7675116_-_TTTTT	TP53	p.165-166:-/KX	frameshift_variant	NA	1.0233
				mis_chr17_7675116_A_T	TP53	p.S166T	missense_variant	COSM45336	1.0465
				mis_chr9_21971112_G_A	CDKN2A	p.H83Y	missense_variant	COSM99723	0.8372
	2016-09-21	8	C1D7	del_chr7_55174772_GGAATTAAGAGAAGC_-	EGFR	p.745-750:KELREA/K	inframe_deletion	COSM6223	0.1316
	2017-01-11	120	C6D1	No call for this sample	NA	NA	NA	NA	NA
	2017-07-26	316	C13D1	No call for this sample	NA	NA	NA	NA	0.7192
	2018-01-10	484	C19D1	No call for this sample	NA	NA	NA	NA	NA
	2018-05-30	624	C24D1	No call for this sample	NA	NA	NA	NA	NA
	2018-08-22	708	C27D1	No call for this sample	NA	NA	NA	NA	NA
LAT009	2016-09-27	-36	Pre	mis_chr7_55181378_C_T	EGFR	p.T790M	missense_variant	COSM6240	47.4719
	2017-02-22	112	ReTxC5D1	mis_chr7_55181378_C_T	EGFR	p.T790M	missense_variant	COSM6240	51.1017
	2017-05-17	196	ReTxC8D1	mis_chr7_55181378_C_T	EGFR	p.T790M	missense_variant	COSM6240	47.8811
	2017-08-09	280	ReTxC11D1	mis_chr7_55181378_C_T	EGFR	p.T790M	missense_variant	COSM6240	47.3171
	2018-01-23	447	ReTxC17D1	mis_chr7_55181378_C_T	EGFR	p.T790M	missense_variant	COSM6240	46.5000
	2018-06-13	588	ReTxC22D1	mis_chr7_55181378_C_T	EGFR	p.T790M	missense_variant	COSM6240	47.6902
	2018-09-05	672	ReTxC25D1	mis_chr7_55181378_C_T	EGFR	p.T790M	missense_variant	COSM6240	48.0000
LAT010	2016-11-16	0	C1D0	amplification_EGFR	EGFR	NA	NA	NA	NA
				del_chr7_55174773_GAATTAAGAGAAGCA_-	EGFR	p.746-750:ELREA/-	inframe_deletion	COSM6225	48.9390
				mis_chr17_7670700_G_A	TP53	p.R337C	missense_variant	COSM117591	13.6340
	2016-11-23	7	C1D7	del_chr7_55174773_GAATTAAGAGAAGCA_-	EGFR	p.746-750:ELREA/-	inframe_deletion	COSM6225	1.7573
				mis_chr17_7670700_G_A	TP53	p.R337C	missense_variant	COSM117591	0.5616
	2017-04-05	140	C6D1	No call for this sample	NA	NA	NA	NA	NA
	2017-08-23	280	C11D1	No call for this sample	NA	NA	NA	NA	NA
	2017-10-18	336	C13D1	del_chr7_55174773_GAATTAAGAGAAGCA_-	EGFR	p.746-750:ELREA/-	inframe_deletion	COSM6225	18.3166
				mis_chr17_7670700_G_A	TP53	p.R337C	missense_variant	COSM117591	2.9146
	2018-01-10	420	C16D1	amplification_EGFR	EGFR	NA	NA	NA	NA
				del_chr7_55174773_GAATTAAGAGAAGCA_-	EGFR	p.746-750:ELREA/-	inframe_deletion	COSM6225	88.6125
				mis_chr17_7670700_G_A	TP53	p.R337C	missense_variant	COSM117591	84.9125
	2018-03-26	495	C18D20	del_chr7_55174773_GAATTAAGAGAAGCA_-	EGFR	p.746-750:ELREA/-	inframe_deletion	COSM6225	19.9160
				mis_chr17_7670700_G_A	TP53	p.R337C	missense_variant	COSM117591	16.2185
	2018-05-09	539	Off	amplification_EGFR	EGFR	NA	NA	NA	NA
				amplification_ERBB2	ERBB2	NA	NA	NA	NA
				del_chr7_55174773_GAATTAAGAGAAGCA_-	EGFR	p.746-750:ELREA/-	inframe_deletion	COSM6225	73.5313
				mis_chr17_7670700_G_A	TP53	p.R337C	missense_variant	COSM117591	40.5875
	2018-06-11	572	Off	amplification_EGFR	EGFR	NA	NA	NA	NA
				del_chr7_55174773_GAATTAAGAGAAGCA_-	EGFR	p.746-750:ELREA/-	inframe_deletion	COSM6225	75.2250
				mis_chr17_7670700_G_A	TP53	p.R337C	missense_variant	COSM117591	48.9125
LAT011	2016-09-28	-56	Pre	del_chr7_55174772_GGAATTAAGAGAAGC_-	EGFR	p.745-750:KELREA/K	inframe_deletion	COSM6223	0.6333
				mis_chr9_21970917_C_T	CDKN2A	p.A148T	missense_variant	COSM3774361	51.2000
	2016-11-23	0	C1D0	del_chr7_55174772_GGAATTAAGAGAAGC_-	EGFR	p.745-750:KELREA/K	inframe_deletion	COSM6223	0.2174
				mis_chr9_21970917_C_T	CDKN2A	p.A148T	missense_variant	COSM3774361	53.4783
	2016-11-30	7	C1D7	mis_chr9_21970917_C_T	CDKN2A	p.A148T	missense_variant	COSM3774361	49.9573
	2016-12-21	28	C2D1	mis_chr9_21970917_C_T	CDKN2A	p.A148T	missense_variant	COSM3774361	47.8182
	2017-08-02	252	C10D1	mis_chr9_21970917_C_T	CDKN2A	p.A148T	missense_variant	COSM3774361	52.3214
	2017-12-20	392	C15D1	del_chr7_55174772_GGAATTAAGAGAAGC_-	EGFR	p.745-750:KELREA/K	inframe_deletion	COSM6223	0.1807
				mis_chr9_21970917_C_T	CDKN2A	p.A148T	missense_variant	COSM3774361	49.8193
	2018-01-16	419	ReTxC1D7	mis_chr9_21970917_C_T	CDKN2A	p.A148T	missense_variant	COSM3774361	52.1514
	2018-03-07	469	ReTxC3D1	del_chr7_55174772_GGAATTAAGAGAAGC_-	EGFR	p.745-750:KELREA/K	inframe_deletion	COSM6223	6.3659
				mis_chr9_21970917_C_T	CDKN2A	p.A148T	missense_variant	COSM3774361	47.7561
	2018-05-30	553	Off	amplification_ERBB2	ERBB2	NA	NA	NA	NA
				del_chr7_55174772_GGAATTAAGAGAAGC_-	EGFR	p.745-750:KELREA/K	inframe_deletion	COSM6223	43.9375
				mis_chr9_21970917_C_T	CDKN2A	p.A148T	missense_variant	COSM3774361	15.1500
LAT013	2016-12-07	0	C1D0	mis_chr17_7673802_C_A	TP53	p.R273L	missense_variant	COSM1640828	15.2375
				mis_chr7_55174774_AATTAAGAGAAGCAACATCTC_TCT	EGFR	p.ELREATSP746-753VS	protein_altering_variant	NA	30.2000
				mis_chr7_55174775_A_C	EGFR	p.E746D	missense_variant	NA	0.3000
				mis_chr7_55181378_C_T	EGFR	p.T790M	missense_variant	COSM6240	10.2938
	2016-12-14	7	C1D7	mis_chr17_7673802_C_A	TP53	p.R273L	missense_variant	COSM1640828	3.1194
				mis_chr7_55174774_AATTAAGAGAAGCAACATCTC_TCT	EGFR	p.ELREATSP746-753VS	protein_altering_variant	NA	6.1343
				mis_chr7_55181378_C_T	EGFR	p.T790M	missense_variant	COSM6240	1.6642
	2017-02-01	56	C3D1	mis_chr17_7673802_C_A	TP53	p.R273L	missense_variant	COSM1640828	0.2632
	2017-03-01	84	C4D1	mis_chr17_7673802_C_A	TP53	p.R273L	missense_variant	COSM1640828	0.5344
				mis_chr7_55174774_AATTAAGAGAAGCAACATCTC_TCT	EGFR	p.ELREATSP746-753VS	protein_altering_variant	NA	0.2290
	2017-03-29	112	C5D1	mis_chr17_7673802_C_A	TP53	p.R273L	missense_variant	COSM1640828	0.8943
				mis_chr7_55174774_AATTAAGAGAAGCAACATCTC_TCT	EGFR	p.ELREATSP746-753VS	protein_altering_variant	NA	2.1545
	2017-05-24	168	C7D1	amplification_MET	MET	NA	NA	NA	NA
				mis_chr17_7673802_C_A	TP53	p.R273L	missense_variant	COSM1640828	2.5676
				mis_chr7_55174774_AATTAAGAGAAGCAACATCTC_TCT	EGFR	p.ELREATSP746-753VS	protein_altering_variant	NA	3.7297
				mis_chr7_55191822_T_G	EGFR	p.L858R	missense_variant	COSM6224	0.4865
	2017-06-21	196	C8D1	amplification_MET	MET	NA	NA	NA	NA
				mis_chr17_7673802_C_A	TP53	p.R273L	missense_variant	COSM1640828	4.9722

				mis_chr7_55174774_AATTAAGAGAAGCAACATCTC_TCT	EGFR	p.ELREATSP746-753VS	protein_altering_variant	NA	7.0139
				mis_chr7_55181378_C_T	EGFR	p.T790M	missense_variant	COSM6240	0.1250
LAT014	2016-12-28	0	C1D0	amplification_EGFR	EGFR	NA	NA	NA	NA
				mis_chr17_7675224_G_C	TP53	p.L130V	missense_variant	COSM437616	5.1646
				mis_chr7_55191822_T_G	EGFR	p.L858R	missense_variant	COSM6224	27.3560
	2017-01-04	7	C1D7	mis_chr17_7675224_G_C	TP53	p.L130V	missense_variant	COSM437616	2.0769
				mis_chr7_55191822_T_G	EGFR	p.L858R	missense_variant	COSM6224	11.1846
	2017-01-25	28	C2D1	mis_chr17_7675224_G_C	TP53	p.L130V	missense_variant	COSM437616	0.9328
				mis_chr7_55191822_T_G	EGFR	p.L858R	missense_variant	COSM6224	4.3843
	2017-03-22	84	C4D1	mis_chr17_7675224_G_C	TP53	p.L130V	missense_variant	COSM437616	1.1170
				mis_chr7_55191822_T_G	EGFR	p.L858R	missense_variant	COSM6224	9.8803
	2017-05-17	140	C6D1	amplification_EGFR	EGFR	NA	NA	NA	NA
				mis_chr17_7675224_G_C	TP53	p.L130V	missense_variant	COSM437616	4.2821
				mis_chr7_55191822_T_G	EGFR	p.L858R	missense_variant	COSM6224	36.5000
	2017-10-04	280	C11D1	amplification_EGFR	EGFR	NA	NA	NA	NA
				amplification_KRAS	KRAS	NA	NA	NA	
				mis_chr17_7675224_G_C	TP53	p.L130V	missense_variant	COSM437616	8.5377
				mis_chr7_55191822_T_G	EGFR	p.L858R	missense_variant	COSM6224	49.6855
	2017-11-01	308	C12D1	amplification_EGFR	EGFR	NA	NA	NA	NA
				amplification_KRAS	KRAS	NA	NA	NA	
				mis_chr17_7675224_G_C	TP53	p.L130V	missense_variant	COSM437616	6.6591
				mis_chr7_55191822_T_G	EGFR	p.L858R	missense_variant	COSM6224	45.2614
	2018-05-04	492	Off	mis_chr17_7675224_G_C	TP53	p.L130V	missense_variant	COSM437616	0.3250
				mis_chr7_55191822_T_G	EGFR	p.L858R	missense_variant	COSM6224	0.3063
	2018-07-11	560	Off	mis_chr17_7675224_G_C	TP53	p.L130V	missense_variant	COSM437616	6.2750
				mis_chr7_55191822_T_G	EGFR	p.L858R	missense_variant	COSM6224	11.5000
	2018-08-31	611	Off	amplification_MET	MET	NA	NA	NA	NA
				mis_chr17_7675224_G_C	TP53	p.L130V	missense_variant	COSM437616	14.6296
				mis_chr7_55191822_T_G	EGFR	p.L858R	missense_variant	COSM6224	25.3588
	2018-09-17	628	Off	amplification_MET	MET	NA	NA	NA	NA
				mis_chr17_7675224_G_C	TP53	p.L130V	missense_variant	COSM437616	51.0125
				mis_chr7_55191822_T_G	EGFR	p.L858R	missense_variant	COSM6224	54.6188
	2018-10-26	657	Off	amplification_MET	MET	NA	NA	NA	NA
				mis_chr17_7675224_G_C	TP53	p.L130V	missense_variant	COSM437616	72.1000
				mis_chr7_55191822_T_G	EGFR	p.L858R	missense_variant	COSM6224	65.0563
LAT015	2017-01-18	-21	Pre	No call for this sample	NA	NA	NA	NA	NA
	2017-02-06	2	Pre	No call for this sample	NA	NA	NA	NA	NA
	2017-02-15	7	ReTxC1D7	mis_chr7_55181378_C_T	NA	NA	NA	NA	NA
	2017-04-05	56	ReTxC3D1	No call for this sample	NA	NA	NA	NA	NA
	2017-05-31	112	ReTxC5D1	mis_chr7_55191822_T_G	NA	NA	NA	NA	NA
LAT016	2017-02-08	0	C1D0	del_chr7_55174772_GGAATTAAGAGAAGC_-	EGFR	p.745-750:KELREA/K	inframe_deletion	COSM6223	4.9416
				mis_chr17_7675143_C_A	TP53	p.V157F	missense_variant	COSM131483	2.6070
				mis_chr7_55181378_C_T	EGFR	p.T790M	missense_variant	COSM6240	1.1187
	2017-02-15	7	C1D7	No call for this sample	NA	NA	NA	NA	NA
	2017-05-30	11	C5D1	No call for this sample	NA	NA	NA	NA	NA
	2017-11-15	280	C11D1	mis_chr7_55174035_G_A	EGFR	p.V726M	missense_variant	NA	0.4321
	2018-01-09	335	C13D1	No call for this sample	NA	NA	NA	NA	NA
	2018-04-04	420	C16D1	No call for this sample	NA	NA	NA	NA	NA
	2018-08-22	560	C21D1	del_chr7_55174772_GGAATTAAGAGAAGC_-	EGFR	p.745-750:KELREA/K	inframe_deletion	COSM6223	2.6006
				mis_chr17_7675143_C_A	TP53	p.V157F	missense_variant	COSM131483	0.7328
	2018-10-17	616	C23D1	No call for this sample	NA	NA	NA	NA	NA
	2018-12-12	672	C25D1	No call for this sample	NA	NA	NA	NA	NA
	2019-02-06	728	C27D1	del_chr7_55174772_GGAATTAAGAGAAGC_-	EGFR	p.745-750:KELREA/K	inframe_deletion	COSM6223	4.6154
				mis_chr17_7675143_C_A	TP53	p.V157F	missense_variant	COSM131483	2.3846
	2019-04-03	784	C29D1	del_chr7_55174772_GGAATTAAGAGAAGC_-	EGFR	p.745-750:KELREA/K	inframe_deletion	COSM6223	0.0813
	2019-05-10	821	Off	amplification_MET	MET	NA	NA	NA	NA
				del_chr7_55174772_GGAATTAAGAGAAGC_-	EGFR	p.745-750:KELREA/K	inframe_deletion	COSM6223	16.1979
				mis_chr17_7675143_C_A	TP53	p.V157F	missense_variant	COSM131483	10.5208
	2019-05-24	835	ReTxC1D0	del_chr7_55174772_GGAATTAAGAGAAGC_-	EGFR	p.745-750:KELREA/K	inframe_deletion	COSM6223	2.3281
				mis_chr17_7675143_C_A	TP53	p.V157F	missense_variant	COSM131483	1.4063
	2019-05-29	840	ReTxC1D7	del_chr7_55174772_GGAATTAAGAGAAGC_-	EGFR	p.745-750:KELREA/K	inframe_deletion	COSM6223	0.4895
				mis_chr17_7675143_C_A	TP53	p.V157F	missense_variant	COSM131483	0.3846
	2019-06-19	861	ReTxC2D1	No call for this sample	NA	NA	NA	NA	NA
	2019-08-14	917	ReTxC4D1	del_chr7_55174772_GGAATTAAGAGAAGC_-	EGFR	p.745-750:KELREA/K	inframe_deletion	COSM6223	0.3412
	2019-09-11	945	ReTxC5D1	del_chr7_55174772_GGAATTAAGAGAAGC_-	EGFR	p.745-750:KELREA/K	inframe_deletion	COSM6223	0.3250
				mis_chr17_7675143_C_A	TP53	p.V157F	missense_variant	COSM131483	0.1375
	2017-03-23	0	C1D0	ins_chr7_55181328_-_AACCCCCAC	EGFR	p.773-774:-/NPH	inframe_insertion	COSM12381	5.5125
				mis_chr17_7673803_G_A	TP53	p.R273C	missense_variant	COSM10659	0.5000
				mis_chr7_55181314_G_C	EGFR	p.V769L	missense_variant	NA	3.3438
	2017-03-29	6	C1D7	ins_chr7_55181328_-_AACCCCCAC	EGFR	p.773-774:-/NPH	inframe_insertion	COSM12381	3.4293
				mis_chr7_55181314_G_C	EGFR	p.V769L	missense_variant	NA	1.8717
	2017-05-17	55	C3D1	ins_chr7_55181328_-_AACCCCCAC	EGFR	p.773-774:-/NPH	inframe_insertion	COSM12381	0.5526
				mis_chr7_55181314_G_C	EGFR	p.V769L	missense_variant	NA	0.3684
				ins_chr7_55181328_-_AACCCCCAC	EGFR	p.773-774:-/NPH	inframe_insertion	COSM12381	6.1673

LAT017	2017-08-09	139	C6D1	mis_chr17_7673803_G_A	TP53	p.R273C	missense_variant	COSM10659	0.8188
				mis_chr7_55181314_G_C	EGFR	p.V769L	missense_variant	NA	3.5105
	2017-09-12	173	ReTxC1D0	amplification_EGFR	EGFR	NA	NA	NA	NA
				ins_chr7_55181328_-_AACCCCCAC	EGFR	p.773-774:-/NPH	inframe_insertion	COSM12381	27.8101
				mis_chr17_7673803_G_A	TP53	p.R273C	missense_variant	COSM10659	2.5194
				mis_chr7_55181314_G_C	EGFR	p.V769L	missense_variant	NA	16.8702
	2017-09-20	181	ReTxC1D7	amplification_EGFR	EGFR	NA	NA	NA	NA
				ins_chr7_55181328_-_AACCCCCAC	EGFR	p.773-774:-/NPH	inframe_insertion	COSM12381	35.9125
				mis_chr17_7673803_G_A	TP53	p.R273C	missense_variant	COSM10659	4.3125
				mis_chr7_55181314_G_C	EGFR	p.V769L	missense_variant	NA	21.6938
LAT019	2017-06-28	0	C1D0	del_chr7_55174772_GGAATTAAGAGAAGC_-	EGFR	p.745-750:KELREA/K	inframe_deletion	COSM6223	0.0633
	2017-07-05	7	C1D7	No call for this sample	NA	NA	NA	NA	NA
	2017-07-26	28	C2D1	mis_chr7_55181378_C_T	EGFR	p.T790M	missense_variant	COSM6240	0.4717
	2017-08-23	56	C3D1	No call for this sample	NA	NA	NA	NA	NA
	2017-11-15	140	C6D1	No call for this sample	NA	NA	NA	NA	NA
	2018-02-07	224	C9D1	No call for this sample	NA	NA	NA	NA	NA
	2018-05-02	308	C12D1	No call for this sample	NA	NA	NA	NA	NA
	2018-07-25	392	C15D1	No call for this sample	NA	NA	NA	NA	NA
	2018-10-17	476	C18D1	No call for this sample	NA	NA	NA	NA	NA
	2019-01-09	560	C21D1	No call for this sample	NA	NA	NA	NA	NA
	2019-04-03	644	C24D1	No call for this sample	NA	NA	NA	NA	NA
LAT021	2017-10-25	-2	Pre	del_chr7_55174772_GGAATTAAGAGAAGC_-	EGFR	p.745-750:KELREA/K	inframe_deletion	COSM6223	5.1625
	2017-11-01	5	C1D5	del_chr7_55174772_GGAATTAAGAGAAGC_-	EGFR	p.745-750:KELREA/K	inframe_deletion	COSM6223	1.5375
	2017-11-29	33	C2D1	No call for this sample	NA	NA	NA	NA	NA
	2018-01-24	89	C4D1	No call for this sample	NA	NA	NA	NA	NA
	2018-03-23	147	C6D1	No call for this sample	NA	NA	NA	NA	NA
	2018-04-18	173	C7D1	No call for this sample	NA	NA	NA	NA	NA
	2018-06-13	229	C9D1	No call for this sample	NA	NA	NA	NA	NA
	2018-08-20	297	Off	No call for this sample	NA	NA	NA	NA	NA
	2018-10-10	348	ReTxC1D7	No call for this sample	NA	NA	NA	NA	NA
	2018-12-26	425	ReTxC4D1	del_chr7_55174772_GGAATTAAGAGAAGC_-	EGFR	p.745-750:KELREA/K	inframe_deletion	COSM6223	0.3172
	2019-01-23	453	ReTxC5D1	del_chr7_55174772_GGAATTAAGAGAAGC_-	EGFR	p.745-750:KELREA/K	inframe_deletion	COSM6223	0.5172
	2019-02-04	465	Off	del_chr7_55174772_GGAATTAAGAGAAGC_-	EGFR	p.745-750:KELREA/K	inframe_deletion	COSM6223	0.4447
	2019-02-13	474	Off	del_chr7_55174772_GGAATTAAGAGAAGC_-	EGFR	p.745-750:KELREA/K	inframe_deletion	COSM6223	7.5156
	2019-03-06	495	Off	del_chr7_55174772_GGAATTAAGAGAAGC_-	EGFR	p.745-750:KELREA/K	inframe_deletion	COSM6223	0.4953
	2019-04-10	530	Off	del_chr7_55174772_GGAATTAAGAGAAGC_-	EGFR	p.745-750:KELREA/K	inframe_deletion	COSM6223	1.1982
	2019-06-08	589	Off	No call for this sample	NA	NA	NA	NA	NA
LAT022	2017-11-29	0	C1D0	del_chr17_7673788_G_-	TP53	p.278:P/X	frameshift_variant	COSM45178	0.2604
				mis_chr7_55174776_TTAAGAGAAG_C	EGFR	p.LREA747-750P	protein_altering_variant	NA	1.0417
	2017-12-06	7	C1D7	No call for this sample	NA	NA	NA	NA	NA
	2017-12-27	28	C2D1	No call for this sample	NA	NA	NA	NA	NA
	2018-01-23	55	C3D1	No call for this sample	NA	NA	NA	NA	NA
	2018-04-18	140	C6D1	No call for this sample	NA	NA	NA	NA	NA
	2018-07-10	223	C9D1	No call for this sample	NA	NA	NA	NA	NA
	2018-10-03	308	C12D1	No call for this sample	NA	NA	NA	NA	NA
	2018-12-28	394	C15D1	No call for this sample	NA	NA	NA	NA	NA
	2019-03-20	476	C18D1	No call for this sample	NA	NA	NA	NA	NA
	2019-06-11	559	C21D1	No call for this sample	NA	NA	NA	NA	NA
	2019-07-10	588	C22D1	No call for this sample	NA	NA	NA	NA	NA
	2019-08-09	618	ReTxC1D0	No call for this sample	NA	NA	NA	NA	NA
	2019-08-14	623	ReTxC1D5	No call for this sample	NA	NA	NA	NA	NA
	2019-09-03	643	ReTxC2D1	No call for this sample	NA	NA	NA	NA	NA
	2019-10-01	671	ReTxC3D1	No call for this sample	NA	NA	NA	NA	NA
LAT023	2017-12-20	-7	Pre	del_chr17_7676149_CCACGGG_-	TP53	p.72-74:PVA/X	frameshift_variant	NA	2.2951
				mis_chr7_55174774_AATTAAGAGAAGCA_CGCCG	EGFR	p.ELREA746-750AP	protein_altering_variant	NA	7.5410
	2017-12-27	0	C1D0	del_chr17_7676149_CCACGGG_-	TP53	p.72-74:PVA/X	frameshift_variant	NA	1.5816
				mis_chr7_55174774_AATTAAGAGAAGCA_CGCCG	EGFR	p.ELREA746-750AP	protein_altering_variant	NA	4.4133
	2018-01-03	7	C1D7	No call for this sample	NA	NA	NA	NA	NA
	2018-02-20	55	C3D1	No call for this sample	NA	NA	NA	NA	NA
	2018-05-16	140	C6D1	No call for this sample	NA	NA	NA	NA	NA
	2018-08-07	223	C9D1	No call for this sample	NA	NA	NA	NA	NA
	2018-10-31	308	C12D1	No call for this sample	NA	NA	NA	NA	NA
	2019-01-23	392	C15D1	No call for this sample	NA	NA	NA	NA	NA
	2019-04-17	476	C18D1	No call for this sample	NA	NA	NA	NA	NA
	2019-07-09	559	C21D1	No call for this sample	NA	NA	NA	NA	NA
	10/3/19	645	C24D1	del_chr17_7676149_CCACGGG_-	TP53	p.72-74:PVA/X	frameshift_variant	NA	0.4878
				mis_chr7_55174774_AATTAAGAGAAGCA_CGCCG	EGFR	p.ELREA746-750AP	protein_altering_variant	NA	2.9268
LAT024	2017-11-29	-43	Pre	No call for this sample	NA	NA	NA	NA	NA
	2018-01-10	-1	Pre	del_chr7_55174773_GAATTAAGAGAAGCA_-	EGFR	p.746-750:ELREA/-	inframe_deletion	COSM6225	0.3155
	2018-01-11	0	C1D0	del_chr7_55174773_GAATTAAGAGAAGCA_-	EGFR	p.746-750:ELREA/-	inframe_deletion	COSM6225	0.1025
	2018-01-17	6	C1D7	No call for this sample	NA	NA	NA	NA	NA
	2018-02-14	34	C2D1	No call for this sample	NA	NA	NA	NA	NA
	2018-03-07	55	C3D1	No call for this sample	NA	NA	NA	NA	NA
	2018-05-30	139	C6D1	No call for this sample	NA	NA	NA	NA	NA

	2018-08-22	223	C9D1	No call for this sample	NA	NA	NA	NA	NA
	2018-11-14	307	C12D1	No call for this sample	NA	NA	NA	NA	NA
	2019-02-06	391	C15D1	No call for this sample	NA	NA	NA	NA	NA
LAT025	2018-04-11	-14	Pre	No call for this sample	NA	NA	NA	NA	NA
	2018-04-25	0	C1D0	del_chr7_55174772_GGAATTAAGAGAAGC_-	EGFR	p.745-750:KELREA/K	inframe_deletion	COSM6223	0.0207
	2018-05-02	7	C1D7	del_chr7_55174772_GGAATTAAGAGAAGC_-	EGFR	p.745-750:KELREA/K	inframe_deletion	COSM6223	0.0391
	2018-05-23	28	C2D1	No call for this sample	NA	NA	NA	NA	NA
	2018-06-20	56	C3D1	No call for this sample	NA	NA	NA	NA	NA
	2018-09-12	140	C6D1	No call for this sample	NA	NA	NA	NA	NA
	2018-12-05	224	C9D1	No call for this sample	NA	NA	NA	NA	NA
	2019-01-04	254	ReTxC1D0	No call for this sample	NA	NA	NA	NA	NA
	2019-01-30	280	ReTxC2D1	del_chr17_7674280_TCAG_-	TP53	p.227-228:SD/X	frameshift_variant	NA	0.0858
				mis_chr12_25245351_C_A	KRAS	p.G12C	missense_variant	COSM516	0.5687
	2019-02-27	308	ReTxC3D1	del_chr17_7674280_TCAG_-	TP53	p.227-228:SD/X	frameshift_variant	NA	0.1681
				mis_chr12_25245351_C_A	KRAS	p.G12C	missense_variant	COSM516	2.2794
	2019-03-15	324	Off	No call for this sample	NA	NA	NA	NA	NA
LAT026	2018-05-02	-36	Pre	No call for this sample	NA	NA	NA	NA	NA
	2018-06-07	0	C1D0	No call for this sample	NA	NA	NA	NA	NA
	2018-06-13	6	C1D7	No call for this sample	NA	NA	NA	NA	NA
	2018-07-05	28	C2D1	mis_chr10_121498499_C_A	FGFR2	p.Q557H	missense_variant	NA	0.2703
	2018-08-01	55	C3D1	No call for this sample	NA	NA	NA	NA	NA
	2018-10-31	146	C6D1	No call for this sample	NA	NA	NA	NA	NA
	2018-12-19	195	C8D1	No call for this sample	NA	NA	NA	NA	NA
	2019-03-13	279	C11D1	No call for this sample	NA	NA	NA	NA	NA
	2019-06-05	363	C14D1	No call for this sample	NA	NA	NA	NA	NA
	2019-06-17	375	Off	No call for this sample	NA	NA	NA	NA	NA
	2019-07-03	391	Off	No call for this sample	NA	NA	NA	NA	NA
	2019-08-07	426	ReTxC1D0	No call for this sample	NA	NA	NA	NA	NA
	2019-08-14	433	ReTxC1D7	No call for this sample	NA	NA	NA	NA	NA
	2019-09-04	454	ReTxC2D1	No call for this sample	NA	NA	NA	NA	NA
	2019-10-02	482	ReTxC3D1	No call for this sample	NA	NA	NA	NA	NA
LAT028	2018-10-24	-2	Pre	del_chr7_55174773_GAATTAAGAGAAGCA_-	EGFR	p.746-750:ELREA/-	inframe_deletion	COSM6225	1.2125
				mis_chr17_7675124_T_C	TP53	p.Y163C	missense_variant	COSM129852	0.5875
	2018-10-26	0	C1D0	del_chr7_55174773_GAATTAAGAGAAGCA_-	EGFR	p.746-750:ELREA/-	inframe_deletion	COSM6225	1.3563
				mis_chr17_7675124_T_C	TP53	p.Y163C	missense_variant	COSM129852	0.5625
	2018-10-31	5	C1D7	del_chr7_55174773_GAATTAAGAGAAGCA_-	EGFR	p.746-750:ELREA/-	inframe_deletion	COSM6225	0.7069
				mis_chr17_7675124_T_C	TP53	p.Y163C	missense_variant	COSM129852	0.3423
	2018-11-21	26	C2D1	No call for this sample	NA	NA	NA	NA	NA
	2018-12-19	54	C3D1	No call for this sample	NA	NA	NA	NA	NA
	2019-01-16	82	C4D1	No call for this sample	NA	NA	NA	NA	NA
	2019-02-13	110	C5D1	del_chr7_55174773_GAATTAAGAGAAGCA_-	EGFR	p.746-750:ELREA/-	inframe_deletion	COSM6225	0.6971
	2019-03-13	138	C6D1	del_chr7_55174773_GAATTAAGAGAAGCA_-	EGFR	p.746-750:ELREA/-	inframe_deletion	COSM6225	1.5044
	2019-04-10	166	C7D1	del_chr7_55174773_GAATTAAGAGAAGCA_-	EGFR	p.746-750:ELREA/-	inframe_deletion	COSM6225	3.0227
	2019-05-08	194	C8D1	del_chr7_55174773_GAATTAAGAGAAGCA_-	EGFR	p.746-750:ELREA/-	inframe_deletion	COSM6225	4.2834
	2019-05-28	214	ReTxC1D0	del_chr7_55174773_GAATTAAGAGAAGCA_-	EGFR	p.746-750:ELREA/-	inframe_deletion	COSM6225	0.1438
	2019-06-05	222	ReTxC1D7	del_chr7_55174773_GAATTAAGAGAAGCA_-	EGFR	p.746-750:ELREA/-	inframe_deletion	COSM6225	5.2000
	2019-06-26	243	ReTxC2D1	amplification_EGFR	EGFR	NA	NA	NA	NA
				del_chr7_55174773_GAATTAAGAGAAGCA_-	EGFR	p.746-750:ELREA/-	inframe_deletion	COSM6225	24.4225
				mis_chr17_7675124_T_C	TP53	p.Y163C	missense_variant	COSM129852	1.2535
	2019-07-16	263	Off	del_chr7_55174773_GAATTAAGAGAAGCA_-	EGFR	p.746-750:ELREA/-	inframe_deletion	COSM6225	0.8563
	2019-07-29	276	Off	del_chr7_55174773_GAATTAAGAGAAGCA_-	EGFR	p.746-750:ELREA/-	inframe_deletion	COSM6225	3.5813
	2019-08-23	301	Off	amplification_EGFR	EGFR	NA	NA	NA	NA
				del_chr7_55174773_GAATTAAGAGAAGCA_-	EGFR	p.746-750:ELREA/-	inframe_deletion	COSM6225	27.6750
				mis_chr17_7675124_T_C	TP53	p.Y163C	missense_variant	COSM129852	2.2000
